# Supplementary material for: The Bond Strength and Antibacterial Activity of the Universal Dentin Bonding System: A Systematic Review and Meta-Analysis
Source: Microorganisms. 2021 Jun 6;9(6):1230. doi: 10.3390/microorganisms9061230 (PMC8227198; doi:10.3390/microorganisms9061230)
Supplement: Supplementary file 1 [file microorganisms-09-01230-s001.zip › microorganisms-1243840-supplementary.pdf]

Table S1. Search strategy used in Scielo.

**Search strategy**	
# 1	Microbial viability OR Antibacterial OR Antimicrobial OR peptide antibacterial OR Antibacterial activity OR Anti-Infective Agents OR Anti Infective Agents OR Antiinfective Agents OR Microbicides OR Antimicrobial Agents OR Anti-Microbial Agents OR Anti Microbial Agents OR anti-Bacterial Agents OR Anti Bacterial Agents OR Antibacterial Agents OR Biofilm OR Bacterial
# 2	Universal adhesives OR Universal adhesive OR Universal simplified adhesive systems OR Universal Dental Adhesives OR Multipurpose adhesives OR multi-purpose adhesives OR multimode adhesives OR multi-mode adhesives OR universal bonding agent
# 3	#1 and #2

Table S2. Search strategy used in ISI Web of Science.

**Search strategy**	
# 1	Microbial viability OR Antibacterial OR Antimicrobial OR peptide antibacterial OR Antibacterial activity OR Anti-Infective Agents OR Anti Infective Agents OR Antiinfective Agents OR Microbicides OR Antimicrobial Agents OR Anti-Microbial Agents OR Anti Microbial Agents OR anti-Bacterial Agents OR Anti Bacterial Agents OR Antibacterial Agents OR Biofilm OR Bacterial
# 2	Universal adhesives OR Universal adhesive OR Universal simplified adhesive systems OR Universal Dental Adhesives OR Multipurpose adhesives OR multi-purpose adhesives OR multimode adhesives OR multi-mode adhesives OR universal bonding agent
# 3	#1 and #2

Table S3. Search strategy used in SCOPUS.

Search strategy	
# 1	Microbial viability" OR "Antibacterial" OR "Antimicrobial" OR "peptide antibacterial" OR "Antibacterial activity" OR "Anti-Infective Agents" OR "Anti Infective Agents" OR "Antiinfective Agents" OR "Microbicides" OR "Antimicrobial Agents" OR "Anti-Microbial Agents" OR "Anti Microbial Agents" OR "anti-Bacterial Agents" OR "Anti Bacterial Agents" OR "Antibacterial Agents" OR "Biofilm" OR "Bacterial"
# 2	"universal adhesives" OR "Universal adhesive" OR "Universal simplified adhesive systems" OR "Universal Dental Adhesives" OR "Multipurpose adhesives" OR "multi-purpose adhesives" OR "multimode adhesives" OR "multi-mode adhesives" OR "universal bonding agent"
# 3	#1 and #2

Table S4. Search strategy used in EMBASE.

Search strategy	
# 1	'Microbial viability' OR 'Antibacterial' OR 'Antimicrobial' OR 'peptide antibacterial' OR 'Antibacterial activity' OR 'Anti-Infective Agents' OR 'Anti Infective Agents' OR 'Antiinfective Agents' OR 'Microbicides' OR 'Antimicrobial Agents' OR 'Anti-Microbial Agents' OR 'Anti Microbial Agents' OR 'anti-Bacterial Agents' OR 'Anti Bacterial Agents' OR 'Antibacterial Agents' OR 'Biofilm' OR 'Bacterial'
# 2	'universal adhesives' OR 'Universal adhesive' OR 'Universal simplified adhesive systems' OR 'Universal Dental Adhesives' OR 'Multipurpose adhesives' OR 'multi-purpose adhesives' OR 'multimode adhesives' OR 'multi-mode adhesives' OR 'universal bonding agent'
# 3	#1 and #2

Table S5. Excluded studies

Study	Reason for exclusion
**Maryoosh, 2020**	The presence of 0 values in Agar diffusion test which cannot be analyzed
**Almaroof, 2017**	The presence of 0 values in Agar diffusion test which cannot be analyzed, and for dentin push-out bond strength there was not enough study group for comparison
**Bosso André, 2017**	The presence of 0 values in Agar diffusion test which cannot be analyzed
**Boutsiouki, 2019**	There was not enough study group for comparison
**Brambilla, 2017**	There was not enough study group for comparison
**Cangul, 2020**	The antibacterial agent is not included in the adhesive
**Bosso André, 2015**	For antibacterial activity, data was not in mean and SD
**Peng, 2020**	The antibacterial agent is not included in the adhesive
**Cha, 2016**	For the antibacterial activity, SD could not be obtained. The antibacterial agent is not included in the adhesive
**Barros Silva, 2021**	Composite disks were used as a bonding substrate
**Atalayin, 2018**	The presence of 0 values in Agar diffusion test which cannot be analyzed
**Zhang, 2020**	The antibacterial agent is not included in the adhesive
**Kim, 2017**	The antibacterial agent is not included in the adhesive
